# Hydroxyhydroquinone impairs fat utilization in mice by reducing nitric oxide availability

**DOI:** 10.1007/s12576-018-0605-9

**Published:** 2018-03-23

**Authors:** Keiko Ishida, Koichi Misawa, Masaki Yamamoto, Akira Shimotoyodome

**Affiliations:** 1 Biological Science Laboratories, Kao Corporation, 2606 Akabane, Ichikai-machi, Haga-gun, Tochigi, 321-3497 Japan; 2 Health Care Food Research Laboratories, Kao Corporation, 2-1-3 Bunka, Sumida, Tokyo 131-8501 Japan

**Keywords:** Fat metabolism, Hydroxyhydroquinone, Liver, Mice, Nitric oxide

## Abstract

Habitual consumption of chlorogenic acid compounds (CGAs) from coffee increases fat catabolism and reduces body fat; however, the contribution of roasted coffee remains unclear. Hydroxyhydroquinone (HHQ) impairs the vasodilatory and antihypertensive effects of CGAs by reducing nitric oxide (NO) bioavailability. Since HHQ also reduces fat catabolism, we hypothesized that HHQ does so by decreasing NO availability. Therefore, we investigated the effect of HHQ on energy metabolism in KKAy mice. In HHQ-treated mice, fat oxidation was significantly low and dose-dependent, serum and urinary hydrogen peroxide were high, and plasma NO metabolites and S-nitrosylated liver proteins were low. In HHQ-treated mouse hepatocytes, the palmitate-induced increase in cellular oxygen consumption was negatively affected, and HHQ or L-NAME reduced cellular fatty acid utilization. In conclusion, HHQ can impair fat utilization by reducing NO availability in mice. Protein S-nitrosylation reduction in liver cells after HHQ consumption may be associated with impaired fatty acid oxidation.

**Electronic supplementary material:**

The online version of this article (10.1007/s12576-018-0605-9) contains supplementary material, which is available to authorized users.

## Introduction

Coffee is a popular beverage worldwide and has been consumed for many years because of its attractive flavor and physiological effects. It is also one of the best-documented foods with respect to epidemiological effects [1]. Coffee consumption is linked to reduced risks of type 2 diabetes [2–4], liver cirrhosis [5, 6], liver and colorectal cancer [7–9], and death associated with inflammatory and cardiovascular diseases [10]. The link between coffee consumption and various health benefits may be attributed to its high polyphenol content [11, 12]. Chlorogenic acids (CGAs) and caffeic acid esters of quinic acid are among the most abundant polyphenols in coffee. CGAs in coffee consist of nine main compounds: 5-caffeoylquinic acid (major component), 3-caffeoylquinic acid, 4-caffeoylquinic acid, 3,4-dicaffeoylquinic acid, 3,5-dicaffeoylquinic acid, 4,5-dicaffeoylquinic acid, 3-feruloylquinic acid, 4-feruloylquinic acid, and 5-feruloylquinic acid (minor components). A single cup of coffee contains 70–350 mg of CGAs [13].

In rodents [14] and humans [15, 16], long-term ingestion of CGAs can reduce body fat, particularly visceral fat, and daily ingestion of CGAs increases fat catabolism. Most studies to date have focused on the effects of green coffee extract ingestion [17, 18]. However, there is limited evidence from epidemiological or interventional studies linking roasted coffee to weight loss or body fat reduction, despite its adequate CGA content. Intake of roasted coffee extract increases the urinary excretion of hydrogen peroxide (H_2_O_2_) in humans. This increase may be attributed to the presence of hydroxyhydroquinone (HHQ; 1,2,4-trihydroxybenzene), a candidate source of reactive oxygen species (ROS) [19], which is formed when coffee beans are roasted [20]. Several studies have demonstrated that HHQ inhibits the antihypertensive effects of CGAs in rodents [21, 22] and humans [23–25] through the production of superoxide anions (O_2_^−^), which can diminish the bioavailability of nitric oxide (NO), an endothelium-derived vasodilator. Our previous study [26] demonstrated that 1-week ingestion of HHQ-reduced coffee led to significantly higher fat utilization in healthy humans than in those that consumed HHQ-containing coffee, which suggests that HHQ decreases fat catabolism in humans. However, the mechanism underlying the reduction of fat catabolism by HHQ remains unclear.

Recent studies have revealed that NO is involved in fatty acid metabolism [27–29]. Furthermore, Doulias et al. [30] showed that NO regulates mitochondrial fatty acid metabolism through post-translational S-nitrosylation of fatty acid oxidation-related enzymes. These findings prompted us to hypothesize that HHQ-derived ROS may inhibit fatty acid metabolism by decreasing NO availability. Therefore, to clarify the mechanism underlying the inhibitory effect of HHQ on fat utilization, we investigated the effect of HHQ on energy metabolism in animals and cell lines.

## Materials and methods

### Materials

All reagents were purchased from Sigma-Aldrich Japan K.K. (Tokyo, Japan) unless otherwise stated.

### Animals

Eight-week-old male KKAy mice (CLEA, Tokyo, Japan) were individually housed in a temperature- and humidity-controlled room (23 ± 2 °C, 55 ± 10% relative humidity) with a 12-h light/12-h dark cycle (lights on at 0700 h). The mice were divided into three groups (*N* = 8 mice/group), and the experiment was conducted over 4 weeks. Either purified water (control group) or water with 100 mg/l (low-HHQ group) or 500 mg/l (high-HHQ group) HHQ (Wako Pure Chemical, Osaka, Japan) was provided as drinking water that was replaced 5 times per week. We determined the dose of HHQ (500 mg/l) in drinking water for the high-HHQ group as the concentration that increased urinary hydrogen peroxide levels of mice to a level similar to that observed after coffee consumption in humans [31, 32]. The mice were fed a vitamin E-deficient powder diet (composed of 10% (w/w) corn oil, 20% casein, 61.5% potato starch, 4% cellulose, 3.5% minerals, 1% vitamins, and < 10 IU vitamin E/kg). The energy content of the diet was 4.16 kcal/g. Individual body weights were recorded weekly, food intake was measured every 2–3 days, and water consumption was measured each time the water was replaced throughout the study. The metabolic rate was measured after 3 weeks by indirect calorimetry as described below. After 4 weeks, the mice were anesthetized by isoflurane (Forane^®^; Abbott, Tokyo, Japan) inhalation. Blood samples were collected from the inferior vena cava. Approximately 0.5 ml of blood was injected into a capillary blood collection tube (CAPIJECT^®^; Terumo, Tokyo, Japan) for serum preparation, whereas another 0.5 ml was injected into a heparinized blood collection tube (CAPIJECT^®^; Terumo) for plasma preparation. After centrifugation (3500×*g*, 10 min, 4 °C), the plasma or serum preparations were stored at − 80 °C until analysis. Urine samples were collected by bladder puncture and stored at − 80 °C until analysis. Tissues were removed, weighed, and stored at − 80 °C until analysis. All animal experiments were conducted in the Experimental Animal Facility of the Kao Tochigi Institute, and protocols were approved by the Kao Corporation Animal Care Committee.

### Indirect calorimetry

Whole-body energy metabolism was assessed by indirect calorimetry using a magnetic-type mass spectrometric calorimeter (Arco-2000; Arco Systems, Chiba, Japan) [33]. Briefly, airflow through the metabolic cages was adjusted to 0.3–0.4 l/min. Mice were acclimated to the metabolic cages for 3 days with free access to experimental diet and water. Locomotor activity was measured using an automated motion system (Actracer-2000; Arco Systems). Data were collected continuously for 12 h (during the light phase) with a settling time of 30 s and a measurement time of 15 s, with room air as reference. Accordingly, the data for each chamber were obtained every 5 min. The respiratory quotient (RQ) and energy substrate (fat and carbohydrate) utilization were calculated from the measured values of oxygen consumption (VO_2_) and carbon dioxide production (VCO_2_).

### Analytical methods

Blood glucose was measured using Accu-Chek Aviva (Roche Diagnostics, Tokyo, Japan) immediately after blood samples were collected. Serum triglyceride (TG) and non-esterified fatty acid (NEFA) levels were determined using specific assay kits (Wako Pure Chemical). After serum and urine samples were filtered through a 10-kDa molecular weight cutoff membrane filtration unit (Amicon Ultra; Merck Millipore, Darmstadt, Germany), serum and urinary H_2_O_2_ concentrations were determined using a quantitative H_2_O_2_ assay kit (Oxis International, Portland, OR, USA). The concentration of plasma NO metabolites (NO_*x*_: NO_2_^−^ and NO_3_^−^) was determined using the method described by Matsumoto et al. [34]. Briefly, 0.03 ml of plasma sample was mixed with 0.03 ml of 100% methanol and centrifuged at 3500×*g* for 10 min at 4 °C. Plasma NO_*x*_ was then determined using an automated NO detector/high-performance liquid chromatography system (ENO20; Eicom, Kyoto, Japan).

### RNA extraction and quantitative PCR (qPCR)

Total RNA was extracted from the liver, thoracic aorta, and soleus muscle tissue samples using the RNeasy Mini kit (Qiagen, Hilden, Germany), RNeasy plus Universal Mini kit (Qiagen), and RNeasy Lipid kit (Qiagen), respectively. For qPCR, cDNA was synthesized using the PrimeScript RT Reagent kit (Takara Bio, Shiga, Japan). qPCR assays were performed on the 7500 Fast Real-Time PCR system (Applied Biosystems, Foster City, CA, USA) using commercially available FAM-labeled TaqMan probes (TaqMan Gene Expression Assays; Applied Biosystems). The expression of each gene was normalized to that of the housekeeping gene encoding ribosomal phosphoprotein P0 (36B4). The genes assessed in this study are listed in Supplemental Table S1.

### Western blot analysis of S-nitrosylated protein

The liver samples were homogenized in 200 µl ice-cold lysis buffer (10 mM Tris–HCl, pH 7.4, 50 mM sodium chloride, 30 mM sodium pyrophosphate, 0.5% Triton X-100, protease inhibitor cocktail, and phosphatase inhibitor cocktail) and incubated on ice for 15 min. The lysate was cleared by centrifugation (16,000×*g*, 15 min, 4 °C). The supernatant was collected in a separate tube, and the proteins were solubilized in 4 × sodium dodecyl sulfate (SDS) sample buffer (Novagen; Merck Millipore). Protein concentrations were determined using a bicinchoninic acid protein assay kit (Thermo Fisher Scientific, Waltham, MA, USA). Samples (20 µg protein/lane) were subjected to SDS-polyacrylamide gel electrophoresis (SDS-PAGE) and transferred to polyvinylidene difluoride membranes. The blots were incubated with mouse anti-S-nitrosocysteine (1:1000) (Abcam, Cambridge, MA, USA) and rabbit anti-β-actin (1:1000) (Cell Signaling Technology, Danvers, MA, USA) antibodies. After washing with 0.1% Tween-20 (TBS/T), the membranes were incubated with horseradish peroxidase (HRP)-conjugated anti-mouse or anti-rabbit IgG (1:1000) (Cell Signaling Technology) secondary antibodies. Since two major S-nitrosocysteine-positive bands were obtained in the analysis, the specific antigenic protein bands were densitometrically quantified after normalization with β-actin using the ChemiDoc XRS imaging system (Bio-Rad, Hercules, CA, USA).

### Primary culture of mouse hepatocytes

Mouse hepatocytes were prepared by the collagenase perfusion method through the postcaval vein [35]. Briefly, 7- to 10-week-old male C56BL/6 J mice (Charles River, Kanagawa, Japan) were anesthetized by isoflurane (Forane^®^; Abbott) inhalation, and the liver was perfused through the postcaval vein with 30 ml of Liver Perfusion Medium (Gibco, Grand Island, NY, USA), followed by 30 ml of Liver Digest Medium (Gibco), at a flow rate of 3–4 ml/min. The perfusion medium was incubated at 37 °C. After perfusion, the liver was removed and immersed in ice-cold Dulbecco’s Modified Eagle’s Medium (DMEM) (Gibco), supplemented with 1% antibiotic–antimycotic mixture (Gibco). The hepatocytes were released by gentle pipetting and then filtered using a 100-µm pore size mesh nylon filter (Corning, NY, USA). The cells were washed thrice with DMEM, supplemented with 1% antibiotic–antimycotic mixture by centrifugation (30×*g*, 3 min, 4 °C), followed by suspension in 10 ml of cell culture medium.

### Measurement of the oxygen consumption rate (OCR) of primary hepatocytes

The OCR of primary hepatocytes was measured using an XF96 Extracellular Flux Analyzer (Seahorse Bioscience, MA, USA). The freshly prepared hepatocytes were seeded at 1.2 × 10^5^ cells/well in XF96 polystyrene cell culture microplates coated with collagen-I in DMEM, containing 10% fetal bovine serum (AusGeneX, Molendinar, Queensland, Australia) and 1% antibiotic–antimycotic mixture. The cells were cultured in 95% air and 5% CO_2_ at 37 °C. After 2–3 h, the cell medium was replaced to remove any unattached cells. Following overnight culture, the cells were treated with HHQ or L-NAME (an inhibitor of nitric oxide synthase [NOS]) for 18 h. On the assay day, the medium was replaced with pre-warmed XF assay medium (DMEM containing 10 mM sodium pyruvate, pH 7.4). The final volume was set to 150 µl/well, and the plate was incubated in a non-CO_2_ incubator (37 °C, 30 min). Six basal measurement cycles were performed before injection of the compound to establish baseline metabolic rates. The XF96 Extracellular Flux Analyzer pneumatically injected 25 μl of 0.7 mM palmitate solution (Pal) or 0.17 mM bovine serum albumin (BSA) solution at pH 7.4 into each culture well at a final concentration of 0.1 mM Pal or 0.02 mM BSA before an 8-loop measurement cycle. Any detectable extracellular flux changes in oxygen were automatically calculated by the XF96 software, with each point representing an average of 7–9 different wells.

### Statistical analyses

Numerical data are expressed as the mean ± SEM. The area under the curve (AUC) was calculated using the trapezoid rule. One-way analysis of variance (ANOVA), followed by Dunnett’s or Bonferroni’s post hoc tests for paired multiple comparisons was used when comparing values among the three groups (GraphPad Prism 6; GraphPad Software, La Jolla, CA, USA). Two-way repeated ANOVA was used to assess changes over time and between groups (GraphPad Prism 6). The Jonckheere’s trend test [36] was conducted to evaluate dose dependency (R version 3.1.2; R Foundation for Statistical Computing, Vienna, Austria). Differences were considered significant when *P* < 0.05.

## Results

### Effect of HHQ on body and liver weights and blood variables

The presence of HHQ in drinking water for 4 weeks did not affect the body or liver weights of mice (Table 1). Both food intake and water consumption decreased with increasing HHQ levels, and consumption levels were significantly lower (*P* < 0.05) in the high-HHQ group than in the control group (Table 1). Blood glucose, TG, and NEFA did not differ among the groups (Table 1).

**Table 1:** Effect of HHQ on body and liver weights and on blood variables

	Control	Low-HHQ	High-HHQ
Body weight (g)	43.18 ± 0.96	43.11 ± 0.83	41.74 ± 0.86
Food intake (g)	290.1 ± 8.4	273.4 ± 4.9	263.1 ± 6.4*
Water consumption (g/day)	18.31 ± 1.11	15.95 ± 0.98	14.34 ± 0.89*
Liver weight (g)	2.54 ± 0.08	2.37 ± 0.09	2.48 ± 0.13
Blood glucose (mg/dl)	474.1 ± 43.6	461.9 ± 35.8	474.3 ± 23.2
Triglycerides (mg/dl)	224.2 ± 15.8	224.3 ± 26.1	250.0 ± 19.8
NEFA (mEq/l)	0.592 ± 0.023	0.549 ± 0.025	0.554 ± 0.014

Values are the mean ± SEM for *N* = 6–8 mice. *P < 0.05 vs control using Dunnett’s post hoc test

### Effect of HHQ on whole-body metabolic rate

**Fig. 1:**
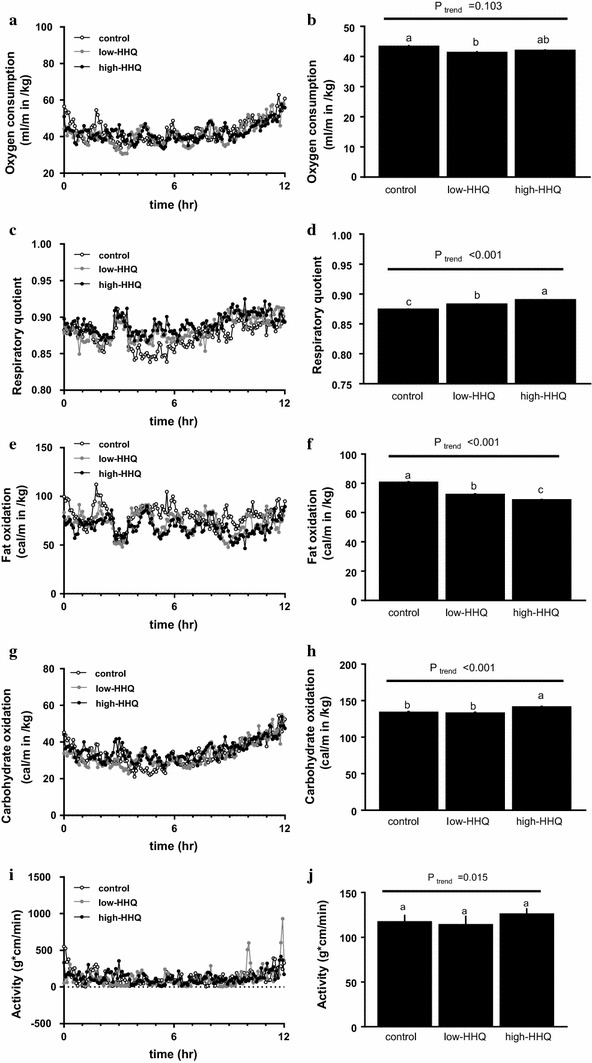
Effect of HHQ on whole-body energy metabolism. **a**, **c**, **e**, **g**, **i** Time-course and **b**, **d**, **f**, **h**, **j** average values of (**a**, **b**) oxygen consumption, **c, d** respiratory quotient, **e**, **f** fat oxidation, **g**, **h** carbohydrate oxidation, and **i**, **j** locomotor activity. The mice were provided with drinking water for 3 weeks prior to measurements. Values are the mean ± SEM of *N* = 8 mice/treatment. *Lower-case letters *above the *bars* (**b**, **d**, **f**, **h**, **j**) represent significant differences between the treatment groups (Bonferroni’s post hoc test). The *P* value for the trend (*P*_trend_) was calculated using the Jonckheere trend test

VO_2_ was significantly lower (*P* < 0.01, Bonferroni’s post hoc test) in the low-HHQ group than in the control group; however, it did not significantly differ between the high-HHQ and control groups (Fig. 1a, b). RQ was significantly different among all three groups (*P* < 0.001, Bonferroni’s post hoc test), with the lowest value in the control group and highest value in the high-HHQ group (Fig. 1c, d). Fat oxidation was also significantly different among all the groups (*P* < 0.001, Bonferroni’s post hoc test) and decreased with increasing HHQ dosage (Fig. 1e, f). Carbohydrate oxidation was significantly higher (*P* < 0.05, Bonferroni’s post hoc test) in the high-HHQ mice than in the control and low-HHQ mice (Fig. 1g, h). Locomotor activity increased with increasing HHQ dosage; however, it did not differ significantly among the groups (Fig. 1i, j).

### Effect of HHQ on serum and urinary H_2_O_2_

**Fig. 2:**
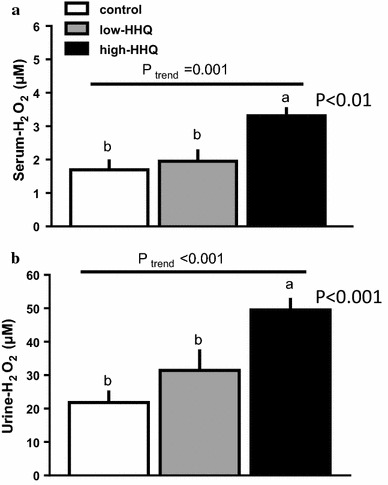
Effect of HHQ on **a** serum and **b** urinary hydrogen peroxide (H_2_O_2_) levels in mice that were provided with drinking water [control: *white bars*, low-HHQ (0.01% HHQ): *gray bars*, and high-HHQ (0.05% HHQ): *black bars*] for 4 weeks. Values are the mean ± SEM of *N* = 7 − 8 mice. *Lower-case letters* above the bars represent significant differences between the treatment groups (*P* < 0.05, one-way ANOVA followed by Bonferroni’s post hoc test), and *P* values of the *black bars* indicate the significance level relative to the control. The *P* value for the trend (*P*_trend_) was calculated using the Jonckheere trend test

Serum (Fig. 2a) and urinary (Fig. 2b) H_2_O_2_ levels increased with increasing HHQ dosage, and they were significantly higher (*P* < 0.01, Bonferroni’s post hoc test) in the high-HHQ group than in the control group.

### Effect of HHQ on gene expression in the liver and soleus muscle tissues

**Fig. 3:**
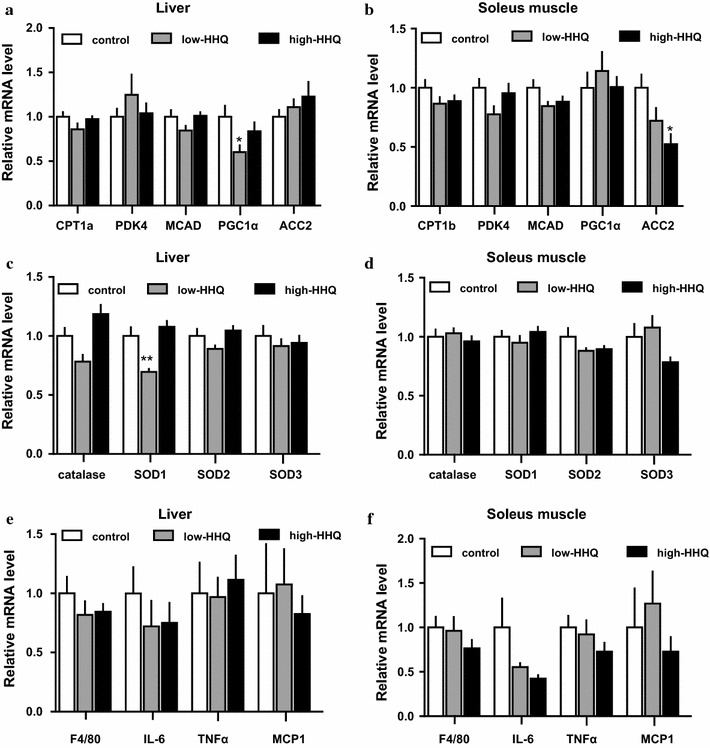
Effect of HHQ on mRNA expression. The mRNA levels of **a**, **b** fat-oxidation related proteins, **c**, **d** antioxidant enzymes, and **e**, **f** inflammation markers in the **a**, **c**, **e** liver and **b**, **d**, **f** soleus muscle of mice that were provided with drinking water [control: *white bars*, low-HHQ (0.01% HHQ): *gray bars*, and high-HHQ (0.05% HHQ): *black bars*] for 4 weeks were determined by qPCR. Each mRNA expression value was normalized to that of the 36B4 mRNA and is shown relative to the control group. Values are the mean ± SEM of *N* = 8 mice. *P < 0.05, **P < 0.01 vs control (Dunnett’s post hoc test)

Among the groups, differences in the mRNA expression of fat-oxidation-related proteins (CPT1a or 1b, PDK4, MCAD, PGC1α, and ACC2) (Fig. 3a, b), antioxidant enzymes (catalase, SOD1, SOD2, and SOD3) (Fig. 3c, d), or inflammation markers (F4/80, IL-6, TNFα, and MCP-1) (Fig. 3e, f) in the liver (Fig. 3a, c, e) and soleus muscle tissues (Fig. 3b, d, f), were not consistently significant. Only the expression levels of liver-PGC1α in the low-HHQ group, soleus-ACC2 in the high-HHQ group, and liver-SOD1 in the low-HHQ group differed significantly from the expression levels in the control group.

### Effects of HHQ on plasma NO metabolites and gene expression of eNOS in the liver and thoracic aorta

**Fig. 4:**
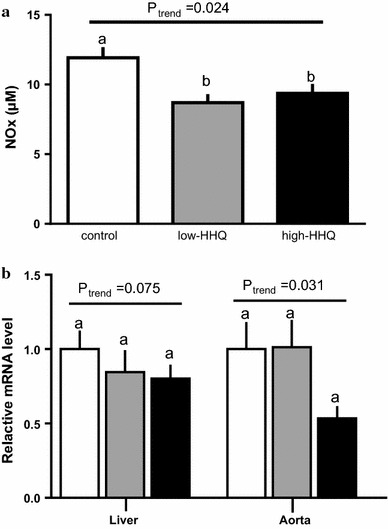
Effects of HHQ on the plasma levels of **a** NO metabolites (NO_*x*_; NO_2_^−^ and NO_3_^−^) and **b** eNOS mRNA expression in the liver and the thoracic aorta. Mice were provided with drinking water [control: *white bars*, low-HHQ (0.01% HHQ): *gray bars*, and high-HHQ (0.05% HHQ): *black bars*] for 4 weeks. Values are the mean ± SEM of *N* = 7 – 8 mice. *Lower-case letters * above the bars represent significant differences between the treatment groups (*P* < 0.05, one-way ANOVA followed by Bonferroni’s post hoc test). The *P* value for the trend (*P*_trend_) was calculated using the Jonckheere trend test

NO metabolites in the plasma were significantly lower (*P* < 0.05, Bonferroni’s post hoc test) in both HHQ-treated groups than in the control group (Fig. 4a). The eNOS mRNA levels in the liver and thoracic aorta of the high-HHQ group tended to be lower than that of the control group (Fig. 4b).

### Effect of HHQ on liver S-nitrosylated protein level

**Fig. 5:**
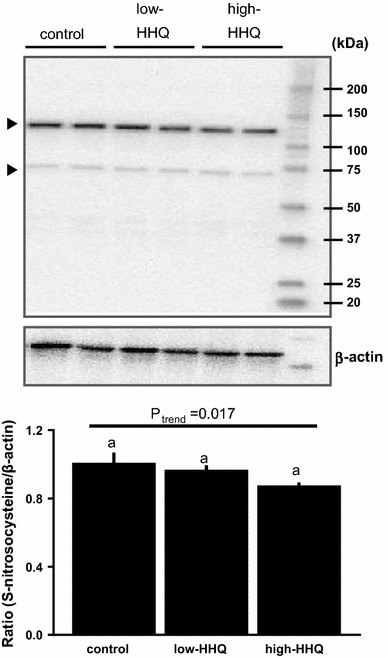
Effect of HHQ on S-nitrosylated proteins in the liver. *Upper panel*: S-nitrosylated protein levels were analyzed by western blotting using an anti-S-nitrosocysteine antibody (*arrowheads*). *Lower panel*: densitometric data were normalized to β-actin levels and shown as values relative to the control. Values are the mean ± SEM of *N* = 8 mice. *Lower-case letters * above the bars represent significant differences between the treatment groups (*P* < 0.05, one-way ANOVA followed by Bonferroni’s post hoc test). The *P* value for the trend (*P*_trend_) was calculated using the Jonckheere trend test

The S-nitrosylated protein levels in liver samples decreased with increasing HHQ concentration; however, the decrease was not significant (*P* = 0.17, Bonferroni’s post hoc test) (Fig. 5).

### Effect of HHQ or L-NAME on fatty acid metabolism in mouse hepatocytes

**Fig. 6:**
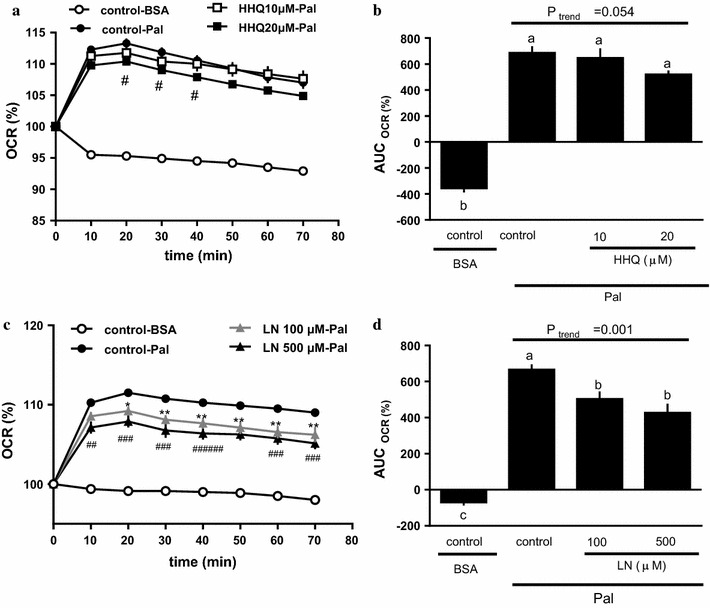
Effect of HHQ and L-NAME on the oxygen consumption rate (OCR) in primary mouse hepatocytes. The energy metabolism of the hepatocytes was measured using an XF96 Extracellular Flux Analyzer. The cells were treated with either HHQ (10 or 20 µM; **a** and **b**) or L-NAME (LN; 100 or 200 µM; **c** and **d**) 18 h before the assay (**a** and **c**). At the start of the assay, either sodium palmitate (Pal) or BSA was added, and the time-course of changes in the OCR was measured (**b** and **d**). The incremental or decremental area under the curve (AUC) was calculated according to the trapezoid rule. Values are the mean ± SEM of *N* = 7 – 9 wells/group. **a** #: ; < 0.05, control-Pal vs HHQ 20 µM-Pal. **c** *P < 0.05, **P < 0.01, control-Pal vs L-NAME 100 µM-Pal; ##: P < 0.01, ###: P < 0.001, control-Pal vs L-NAME 200 µM-Pal (Dunnett’s post hoc test). **b**, **d** *Lower-case letters * above the bars represent significant differences between the treatment groups (*P* < 0.05, one-way ANOVA followed by Bonferroni’s post hoc test). The *P* value for the trend (*P*_trend_) was calculated using the Jonckheere trend test

The OCR increased significantly following the addition of sodium palmitate (control-Pal) to cultured hepatocytes (Fig. 4a, b). The palmitate-induced OCR increase appeared to be negatively affected by increasing HHQ concentration (Fig. 6b); however, the effect was not significant (*P*_trend_ = 0.054, Jonckheere’s trend test) (Fig. 6a, b). L-NAME also regulated the palmitate-induced OCR increase in a dose-dependent manner (Fig. 6d), with the OCR of HHQ-treated mouse hepatocytes being significantly lower than that of control mouse hepatocytes (Fig. 6c, d).

## Discussion

We observed that HHQ consumption increased blood and urinary H_2_O_2_ levels, reduced blood NO metabolites and liver S-nitrosylated protein levels, and decreased whole-body fat utilization in mice. HHQ treatment and NOS inhibition by L-NAME also decreased fatty acid oxidation in mouse primary liver cells. These results agree with our previous finding that HHQ-reduced coffee led to higher fat utilization than HHQ-containing coffee in humans [26], and support our hypothesis that HHQ-derived ROS could impair fatty acid metabolism by decreasing NO bioavailability.

Consumption of HHQ in roasted coffee can increase H_2_O_2_ urinary excretion in humans [19]. In spontaneously hypertensive rats [21, 22] and humans [23–25], HHQ consumption has been found to impair the antihypertensive effect of CGAs associated with increased ROS production and decreased NO availability. The results of this study are in line with previous findings, supporting the observation that HHQ consumption increases whole-body oxidative stress. Our results suggest that not only did HHQ-derived ROS directly decrease NO availability, but also overproduction of ROS after HHQ ingestion resulted in decreased NO production through downregulation of eNOS gene expression.

Interestingly, this study is the first to demonstrate that HHQ consumption can decrease fat metabolism in mice. Previous studies have suggested that oxidative stress is elevated in obesity, diabetes, and non-alcoholic steatohepatitis, and that this elevation possibly leads to fat metabolism suppression [37, 38]. Our results indicated that an increase in oxidative stress suppresses fat catabolism in the context of pathophysiological conditions.

Serviddio et al. [38] proposed that oxidative stress impairs the activity of CPT-1, the rate-limiting enzyme of fatty acid oxidation. Setoyama et al. [39] demonstrated that CPT-1 enzymatic activity could be significantly inhibited by ROS in several human cell types. These findings corroborate our results. A possible explanation for decreased fat utilization after HHQ treatment is that HHQ-derived ROS may interfere with liver CPT-1 activity. Another possible explanation is that oxidative stress following HHQ consumption decreases fat catabolism by reducing NO availability. HHQ-derived superoxide anions rapidly inactivate NO to form peroxynitrite and inhibit NOS activity [40]. Several studies have demonstrated the involvement of NO in regulation of fatty acid metabolism [27–29]. The results of the present study are in agreement with those of previous studies, indicating that HHQ interferes with fatty acid metabolism by decreasing NO availability.

A seminal study by Doulias et al. [30] demonstrated that NO regulates mitochondrial fatty acid oxidation through the reversible activation of critical enzymes by protein S-nitrosylation, which regulates enzymatic activity as well as protein localization and stability [30, 41]. Our results suggest that reduced protein S-nitrosylation in liver cells plays a significant role in decreased fat metabolism after HHQ treatment.

Nevertheless, this study did not identify the altered fat utilization-associated proteins that are differentially S-nitrosylated in the liver after HHQ consumption. Further studies are in progress to identify the target proteins of S-nitrosylation in the liver after HHQ consumption. This study was also unable to determine whether HHQ from roasted coffee reduces fat metabolism in humans, thus warranting further clinical studies.

Although daily consumption of coffee polyphenols, such as CGAs, has been shown to increase fat catabolism and reduce obesity in both rodents [14, 17, 18] and humans [15, 16], whether daily consumption of roasted coffee with an adequate level of CGAs affects obesity remains unclear. We hypothesize that reducing HHQ levels in roasted coffee, while maintaining high CGA levels, may enhance the potential of coffee polyphenols that prevent and decrease obesity.

## Conclusion

This study demonstrated that consumption of HHQ, formed during the roasting of coffee beans, could decrease fat metabolism by increasing oxidative stress and suppressing NO availability in mice. Therefore, the reduction of HHQ levels in roasted coffee may stimulate fat utilization by increasing NO availability. This may have important implications for the development of HHQ-reduced coffee that can potentially prevent or decrease obesity.

### Electronic supplementary material

Below is the link to the electronic supplementary material.

Supplementary material 1 (DOC 28 kb)
