# Supplementary material for: Hydroxyhydroquinone impairs fat utilization in mice by reducing nitric oxide availability
Source: J Physiol Sci. 2018 Mar 23;68(6):855–64. doi: 10.1007/s12576-018-0605-9 (PMC6223984; doi:10.1007/s12576-018-0605-9)
Supplement: Supplementary file 1 — Supplementary material 1 (DOC 28 kb) [file 12576_2018_605_MOESM1_ESM.doc]

Table S1.

Gene symbol Assay ID

Mouse CPT1a Mm01231183_m1

CPT1b Mm00487191_g1

PDK4 Mm01166879_m1

MCAD Mm01323360_g1

PGC1α Mm01208835_m1

ACC2 Mm01204671 m1

Catalase Mm00437992_m1

SOD1 Mm01344233_g1

SOD2 Mm01313000_m1

SOD3 Mm00448831_s1

F4/80 Mm00802529_m1

IL-6 Mm0046190_m1

TNFα Mm00443258_g1

MCP1 Mm00441242_m1

eNOS Mm00435217_m1

36B4 Mm00725448_s1

These pre-designed primers and probe sets were obtained from Applied Biosystems.
